# Chemical Basis of Reactive Oxygen Species Reactivity and Involvement in Neurodegenerative Diseases

**DOI:** 10.3390/ijms20102407

**Published:** 2019-05-15

**Authors:** Fabrice Collin

**Affiliations:** Laboratoire des IMRCP, Université de Toulouse, CNRS UMR 5623, Université Toulouse III-Paul Sabatier, 118 Route de Narbonne, 31062 Toulouse CEDEX 09, France; fabrice.collin@univ-tlse3.fr

**Keywords:** reactive oxygen species, superoxide anion, hydroxyl radical, hydrogen peroxide, hydroperoxides, neurodegenerative diseases, NADPH oxidase, superoxide dismutase

## Abstract

Increasing numbers of individuals suffer from neurodegenerative diseases, which are characterized by progressive loss of neurons. Oxidative stress, in particular, the overproduction of Reactive Oxygen Species (ROS), play an important role in the development of these diseases, as evidenced by the detection of products of lipid, protein and DNA oxidation in vivo. Even if they participate in cell signaling and metabolism regulation, ROS are also formidable weapons against most of the biological materials because of their intrinsic nature. By nature too, neurons are particularly sensitive to oxidation because of their high polyunsaturated fatty acid content, weak antioxidant defense and high oxygen consumption. Thus, the overproduction of ROS in neurons appears as particularly deleterious and the mechanisms involved in oxidative degradation of biomolecules are numerous and complexes. This review highlights the production and regulation of ROS, their chemical properties, both from kinetic and thermodynamic points of view, the links between them, and their implication in neurodegenerative diseases.

## 1. Introduction

Reactive Oxygen Species (ROS) are radical or molecular species whose physical-chemical properties are well-known both on thermodynamic and kinetic points of view. They are produced from molecular oxygen, during the successive 4 steps of 1-electron reduction (reaction (1)). The reaction occurs in particular in the mitochondrial respiratory chain, where 85% of O_2_ is metabolized and where partially reduced O_2_ intermediates are produced in low quantity [1].

(1) $$O_{2}\overset{+e^{-}}{\to }O_{2}^{•-}\overset{+e^{-}(+2H^{+})}{\to }H_{2}O_{2}\overset{+e^{-}}{\to }HO^{•}(+HO^{-})\overset{+e^{-}(+2H^{+})}{\to }2H_{2}O$$

**Figure 1:**
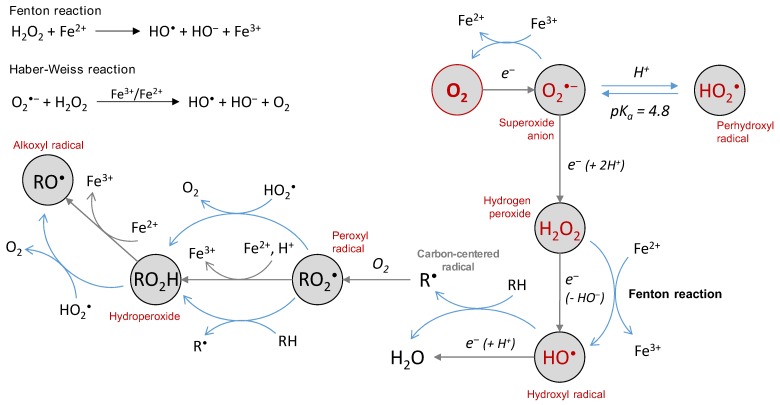
The chemical basis of Reactive Oxygen Species (ROS) generation—primary radical and molecular species are produced by incomplete reduction of molecular oxygen and can further react with an organic substrate to generate substrate-derived ROS. Metal ions are engaged in electron transfer (through metalloenzymes in vivo), but also involved in both Fenton and Haber-Weiss reactions, and in the reduction of hydroperoxide into alkoxyl radical.

The three primary species, i.e., the superoxide anion (O_2_^•‒^), hydrogen peroxide (H_2_O_2_) and the hydroxyl radical (HO^•^), are called reactive oxygen species because they are oxygen-containing compounds with reactive properties. O_2_^•‒^ and HO^•^ are commonly referred to as “free radicals”. They can react with organic substrates and lead to intermediate species able to further produce other ROS. For instance, H atom abstraction by HO^•^ free radicals on a C-H bond leads to a carbon-centered radical, that further reacts rapidly with O_2_ to give a peroxyl radical RO_2_^•^ (Figure 1) [2]. The latter may react with another substrate to give a new carbon-centered radical and a hydroperoxide ROOH, which may decompose into alkoxyl radical RO^•^ in a reaction catalyzed by redox competent metal cations such as iron or copper (as occurring with heme proteins [3]). These “secondary” species are all ROS and share a similarity in structure and reactivity with the three primary species O_2_^•‒^, H_2_O_2_ and HO^•^. Among them, H_2_O_2_ (and hydroperoxides) is a molecular species and is supposed to be less reactive than the other radical short-lived species that are able to react with a range of targets (an exception may apply for O_2_^•‒^). However, its toxicity can be exerted via Fenton reaction in the presence of redox metal ions such as iron or copper (Figure 1), or via Haber–Weiss reaction in the presence of O_2_^•‒^ [4].

## 2. Production of ROS

### 2.1. Production of ROS In Vivo, Regulation and Oxidative Stress

ROS can be deleterious for biomolecules and lead to oxidative damages involved in several pathologies (neurodegenerative diseases, atherosclerosis, cancer and other disorders). However, they play, above all, an important role in homeostasis, cell signalization, regulation of metabolism, or memory formation via DNA methylation [5,6]. As recently reviewed, oxidative stress may be a key modulator in neurodegenerative diseases [7]. In mammalian cells, ROS are essentially produced by enzymes and are from different origins: mainly from the cytoplasmic membrane NADPH oxidase and from the enzyme complex of the mitochondrial respiratory chain, but also from sources of other organelles such as xanthine oxidase (XO), lipo- and cyclo-oxygenase, cytochromes P450 (endoplasmic reticulum) and peroxisomes. NADPH oxidase catalyzes the monoelectronic reduction of molecular oxygen, thus producing O_2_^•‒^ [8,9] that is released either outside the cell (for phagocytic cells) or inside the cell (for non-phagocytic cells) [10]. In mitochondria, ROS are produced during ATP biosynthesis which is accompanied by electron and proton transfers, with molecular oxygen as the final target. Electron leaks, which represent around 1–3% of the total electron production, may occur in complex I (NADH-ubiquinone oxidoreductase) and complex III (ubiquinol-cytochrome c oxidoreductase) of the electron transport chain and leads to the production of O_2_^•‒^ [11]. Because of the high activity of the mitochondrial respiratory chain in aerobic organisms, such a leak is the major source of ROS production in cells, more important than NAPDH oxidase (except during the activation of phagocytic cells) and XO [1]. The latter is a molybdenum enzyme, essentially located in the cytosol, that catalyzes the oxidation of hypoxanthine into xanthine and produces O_2_^•‒^, which might be further converted into H_2_O_2_ by XO (and oxidation of xanthine into uric acid) or by cytosolic Superoxide Dismutase (SOD) [12]. Xanthine oxidase is also able to convert nitrite into nitric oxide, and is thus a potential source of peroxynitrite [13]. Lipoxygenases and cyclooxygenases, which oxidize arachidonic acid into leukotrienes and prostanoids (including thromboxanes and prostaglandins), respectively, are other potential sources of ROS [14,15]. In the endoplasmic reticulum, enzymes belonging to the family of cytochromes P450 play a key role in the metabolism of drugs and other xenobiotics [16]. They reduce molecular oxygen to generate O_2_^•‒^ and H_2_O_2_, the latter being involved in the redox regulation of some essential functions of the endoplasmic reticulum [17].

In mitochondria, O_2_^•‒^ and H_2_O_2_ participate in redox signaling [18], but their production is significantly enhanced during oxidative stress conditions, as, for instance, in response to various diseases or stimuli. Oxidative stress reflects an imbalance between the production of ROS and the action of the antioxidant defense system in charge of their neutralization. They include enzymes, namely SOD that reduce O_2_^•‒^ into H_2_O_2_ [19,20], and catalase, glutathione peroxidases and thioredoxin reductase that regulate levels of H_2_O_2_ by converting it into H_2_O and O_2_ [21,22]. The selenoproteins glutathione peroxidases, among which the most abundant is the cytoplasmic and mitochondrial GPx1 [23], are also able to reduce hydroperoxides into alcohols. In addition to the enzymatic systems of defense, the regulation of the oxidative balance in vivo and the protection against oxidative attacks are also carried out by a myriad of non-enzymatic antioxidant systems, among which some are endogenous (glutathione, bilirubin, coenzyme Q, lipoic acid, melatonin, uric acid, etc.) and other ones are exogenous (α-tocopherol, ascorbic acid, carotenoids, etc.). Thus, under oxidative stress conditions, biomolecules may undergo the attack of ROS and get oxidized. Most of the time, such phenomena are deleterious for cells, but in some case, inducing an overproduction of ROS can help kill cells such as cancer cells [24].

### 2.2. Production of ROS In Vitro

Several commonly used methods are available for producing ROS in vitro, either based on metal-catalyzed production or not, and capable for some to selectively produce ROS. Water radiolysis is one of them and consists of irradiating water with γ-rays of ^60^Co or ^137^Cs (or X-ray). The initial energy deposition leads in situ to the generation of the primary radical and molecular species HO^•^, H^•^, e_aq_^‒^ (solvated electron), H_2_O_2_, H_2_ and H^+^, with well-known radiolytic yields of production [25]. The cumulated amount of ROS produced is directly linked to the radiation dose (expressed in Gy), which is dependent on the time the sample is exposed to the radiation source: the longer the exposure, the higher the radiation dose. Thus, it is easy to modulate the amount of ROS produced. A second advantage lies in the possibility of selecting ROS for a specific attack on a substrate: in aerated solutions ([O_2_] ≈ 2 × 10^−4^ mol L^−1^ in water), O_2_^•‒^, HO^•^ and H_2_O_2_ are generated [26,27], whereas O_2_^•‒^ or HO^•^ are selectively produced (along with H_2_O_2_) in 0.1 M sodium formate aqueous solution [28,29] or N_2_O-saturated water [30,31], respectively. For the diluted solution (below 10^−2^ mol·L^−1^), no direct interaction of radiation with the substrate occurs [25] and the latter is only oxidized by the ROS produced by water radiolysis. The production of ROS in vitro may also be achieved through the xanthine/xanthine oxidase system, an enzymatic way of selectively producing O_2_^•‒^ [32]. The selective production of HO^•^ is usually obtained by the Fenton reaction where Fe^2+^ reduces H_2_O_2_ into HO^•^ and HO^‒^ (Figure 1). In this case, ROS are generated by a metal-catalyzed reaction and the resulting oxidative damages are often site-directed, in particular when biomolecules are able to coordinate metal ions [33]. The same applies when ROS are produced by the Cu^2+^/ascorbate system, able to successively generate O_2_^•‒^, H_2_O_2_ and HO^•^ [34,35,36]. For such systems, and unlike gamma radiolysis, the reaction continues as long as there are reagents, although it can be stopped in some cases [36,37]. The modulation of the production of ROS is more difficult to implement.

## 3. Chemical Properties and Reactivity of ROS

### 3.1. The Superoxide Anion

**Figure 2:**
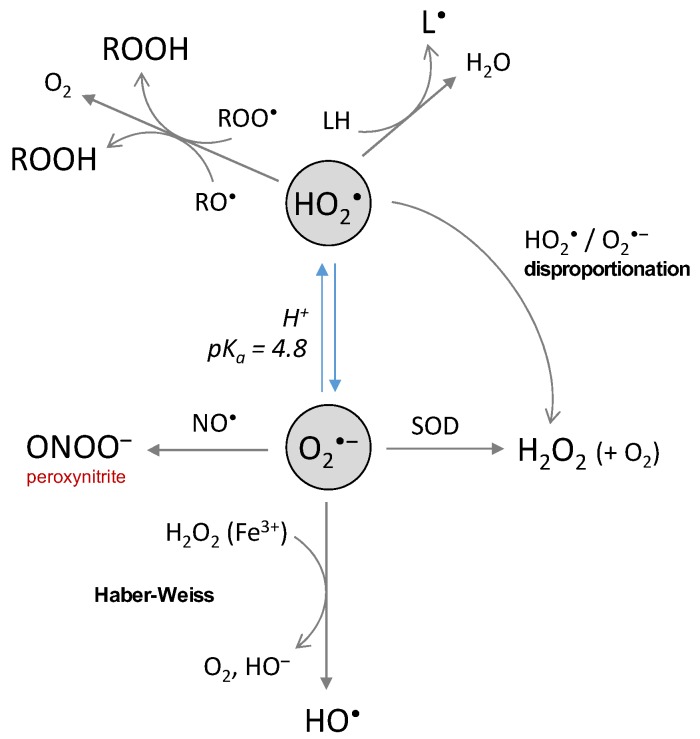
The chemical reactions of the superoxide and the perhydroxyl radicals.

The superoxide anion is generated by the first 1-electron reduction of oxygen. At low pH, it is protonated and called perhydroxyl radical, with pKa(HO_2_^•^/O_2_^•‒^) = 4.8 [38] (Figure 1). There are two redox standard potentials for O_2_^•‒^, showing that it can act as a reductant (E°’(O_2_/O_2_^•‒^) = −0.33 V) or as an oxidant (E°’(O_2_^•‒^/H_2_O_2_) = 0.93 V) [39]. The 1-electron reduction of oxygen is not thermodynamically favored compared to its complete reduction (4 electrons, E°’(O_2_/H_2_O) = 0.81 V). Redox potentials also show that O_2_^•‒^ disproportionation and reduction of H_2_O_2_ by O_2_^•‒^ [40] (Haber–Weiss reaction, Figure 2) are thermodynamically spontaneous reactions.

Despite the relatively high values of its redox potential, O_2_^•‒^ is not a good reductant nor a good oxidant towards most of the biological substrates because of low rate constant values (usually below 10^2^ L·mol^−1^·s^−1^) [38]. Some exception applies as O_2_^•‒^ is able to react with a few favored targets, with the rate constant ranging from 10^5^ to 10^9^ L·mol^−1^·s^−1^ [1]: cytochrome c, ascorbate and SOD (for which O_2_^•‒^ is the substrate). Recently, cytochrome c was used as a probe to demonstrate that O_2_^•‒^ was produced as an intermediate by the system Cu(I)-Aβ/O_2_ [41]. The perhydroxyl radical is more reactive (E°’(HO_2_^•^/H_2_O_2_) = 1.48 V) and able to oxidize polyunsaturated fatty acids such as linoleic, linolenic or arachidonic acids (k = 1.18 × 10^3^, 1.70 × 10^3^ and 3.05 × 10^3^ L·mol^−1^·s^−1^) [42]. It is also engaged in the conversion of the peroxyl radical to hydroperoxide (Figure 2) and then to the alkoxyl radical [43]. The protonated form of O_2_^•‒^ could thus be the reactive one even if it is present at low concentrations at physiological pH. The toxicity of O_2_^•‒^ in a biological context is rather indirect since it is involved in the generation of highly-reactive secondary species. In the Haber–Weiss reaction (Figure 2), O_2_^•‒^ reacts with H_2_O_2_ to produce HO^•^ radicals. The reaction is thermodynamically favored, but not kinetically [40,44,45], and needs to be catalyzed by iron. Disproportionation of perhydroxyl and of perhydroxyl/superoxide radicals (Figure 2) also represent a part of indirect toxicity of the superoxide anion as a potential source of H_2_O_2_. The rate constant is 6 × 10^5^ L·mol^−1^·s^−1^ at pH 7, thus, the reaction is relevant under physiological conditions. The disproportionation of O_2_^•‒^, while thermodynamically spontaneous, is not kinetically favored [38]. Finally, the reaction of O_2_^•‒^ with ^•^NO (k = 1.9 × 10^10^ L·mol^−1^·s^−1^) [46] to generate the highly-reactive peroxynitrite ONOO^‒^ is another reaction conferring an indirect toxicity to O_2_^•‒^, in particular towards DNA, proteins and lipids [47,48]. Peroxynitrite is able to nitrate tyrosine or tryptophan residues, or to oxidize methionine residues [49,50,51].

The reactivity of the superoxide anion does not always lead to a deleterious effect towards biomolecules as it is also able to help to fight against oxidative damages. Recently, Muñoz-Rugeles et al. [52] have shown that the superoxide anion is able to repair oxidized DNA by transferring one electron to the guanosyl radical of a single-stranded DNA. However, such an involvement in unusual chemical processes remains almost unexplored.

### 3.2. The Hydroxyl Radical

The hydroxyl radical is the most powerful oxidant among the ROS, with a potential of E°’(HO^•^/H_2_O) = 2.34 V [39]. At very low pH, HO^•^ converts into its conjugate base O^•‒^ (pKa(HO^•^/O^•‒^) = 11.9), the oxide radical, which is less reactive [53] but not relevant at physiological pH. Reactions of HO^•^ radicals with most substrates are diffusion-controlled (rate constants of 10^10^ L·mol^−1^·s^−1^) as, for example, with biological molecules such as DNA bases, aromatic amino acids, albumin, hemoglobin, linoleate or ascorbate [53,54,55]. Thus, HO^•^ radicals are engaged in fast reactions, with an activation energy close to zero, meaning that they are not able to diffuse, have a very short lifetime (few 10^−6^ s) and free course (few 10^−8^ m), and are weakly selective towards molecular targets. A side consequence of this high reactivity is that the disproportionation of HO^•^ radicals, even if kinetically favored (k ≈ 5 × 10^9^ L·mol^−1^·s^−1^) [54], remains a rather infrequent event in biological conditions, the probability of collision between two hydroxyl radicals being very low.

**Figure 3:**
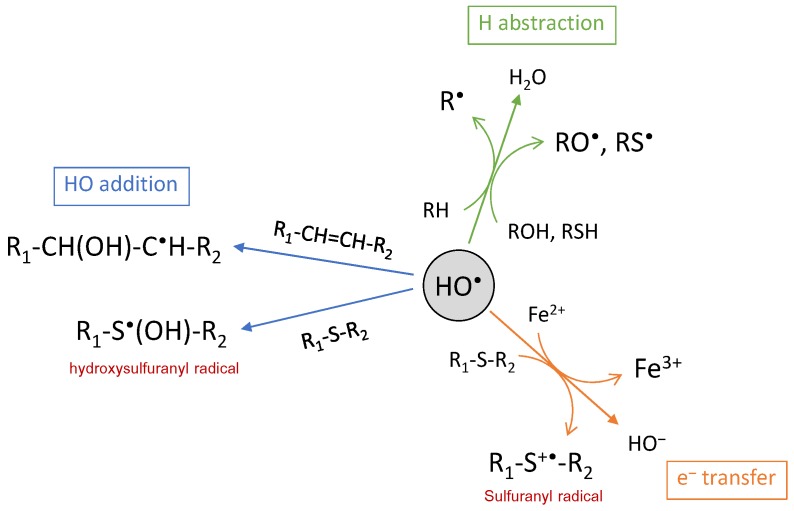
The chemical reactions of the hydroxyl radical.

There are three ways of action for the HO^•^ radical: electron abstraction, hydrogen abstraction and double bond addition (Figure 3). The HO^•^ radical is electrophilic and has a strong affinity for electron-rich sites of molecules, in particular for aromatic or sulfur-containing molecules. This is illustrated by the rate constants of reaction with amino acids, ranging from 10^7^ L·mol^−1^·s^−1^ for Gly to 10^10^ L·mol^−1^·s^−1^ for His, Trp or Cys [53]. Addition reactions are usually faster than H atom abstraction [56], except with Cys where H abstraction from the thiol group is faster [57,58]. HO^•^ addition is commonly involved in biomolecule oxidation, such as oxidation of guanine into 8-oxoguanine [59], of histidine into 2-oxohistidine [60,61] or of tryptophan into N-formylkynurenine and kynurenine [43,62]. Hydroxyl radical addition may also occur on the sulfur atom, in particular, from methionine residue, leading to the hydroxysulfuranyl radical as the intermediate species and, finally, to methionine sulfoxide and sulfone as the end-products [63]. Methionine also undergoes electron abstraction when reacting with HO^•^, thus generating the sulfuranyl radical cation that is able to further evolve. In this case, oxidation leads to irreversible biological damages, contrary to the oxidation of methionine into methionine sulfoxide for which reversibility is ensured through methionine sulfoxide reductases (MsrA and MsrB) [64]. Electron abstraction is also observed with inorganic substrates such as ferrous ions or halides, with high rate constants [55]. The last pathway for HO^•^ reaction is H-abstraction, for which numerous and various biomolecules are targets as, for instance, polyunsaturated fatty acids such as linoleate [65,66] or arachidonate [67,68], sulfur-containing, basic and aromatic amino acid residues from protein and peptides [69,70], or 2-deoxyribose and DNA bases [71]. Most of the time, H abstraction leads to a carbon-centered radical that either further reacts fast with molecular oxygen to generate a peroxyl radical or, in the absence of oxygen, is engaged in a biradical reaction generating a carbon-carbon bond [72]. However, abstraction may also occur on the hydroxyl or thiol functional groups, leading to oxygen- or sulfur-centered radicals [73]. Such mechanisms are observed for protein and peptide cross-linking via bityrosine formation [70,74] or disulfide bridge formation [75].

### 3.3. Hydrogen Peroxide

**Figure 4:**
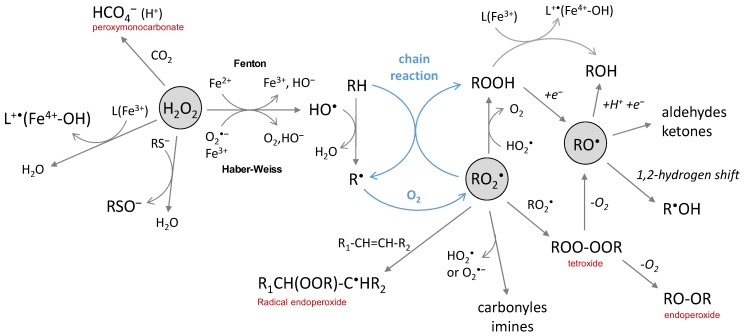
The chemical reactions of hydrogen peroxide, peroxyl and alkoxyl radicals.

Hydrogen peroxide is produced by the two-electron reduction of molecular oxygen. Its conjugate base HOO^‒^ is a strong nucleophile but not relevant at physiological pH because of a high pKa value (pKa(H_2_O_2_/HOO^‒^) = 11.6). H_2_O_2_ is either a reductant or an oxidant in one-electron transfer reactions. The latter is not thermodynamically favored in biological conditions (E°’(O_2_^•‒^/H_2_O_2_) = 0.93 V and E°’(H_2_O_2_/HO^•^) = 0.30 V) [39] but H_2_O_2_ can act as an oxidant if catalysis by metal ions takes place (Fenton and Haber–Weiss reactions). It is rather engaged in two-electron transfer reactions, with a high potential (E°’(H_2_O_2_/H_2_O) = 1.32 V) in physiological conditions. It is more oxidizing than hypochlorous acid and peroxynitrite (E°’(ClO^‒^/Cl^‒^) = 1.28 V and E°’(ONOO^‒^/NO_2_^‒^) = 1.20 V). However, it reacts only poorly with most biological molecules because of a high activation energy barrier, oxidation by H_2_O_2_ being kinetically driven. Thus, the strongest oxidizing power of hydrogen peroxide comes indirectly from its metal-catalyzed conversion into HO^•^ radicals by the Fenton and Haber–Weiss reactions (Figure 4).

In proteins, H_2_O_2_ reacts as a two-electron oxidant towards sulfur-containing residues (cysteine and methionine) but with a low rate constant (k = 2.9 L·mol^−1^·s^−1^ for cysteine) [76]. For thiols, the reaction is exclusive to the thiolate anion, thus the reactivity at physiological pH is dependent on pKa values. It leads to sulfenic acid (RSOH) as the initial product, able to be oxidized one more time by H_2_O_2_ into sulfinic acid (RSO_2_H) or to react with thiols to form disulfides. The highest rate constants are observed for the thiol proteins peroxiredoxins and glutathione peroxidases that react with H_2_O_2_ several orders of magnitude faster (~10^7^ L·mol^−1^·s^−1^) [77]. Such a difference is explained by the polarization of the O-O bond of H_2_O_2_ by hydrogen bonding into the protein that facilitates the electrophilic attack on the thiolate. Pyruvate oxidation in acetate and carbon dioxide by H_2_O_2_ is also biologically relevant because of a rate constant of 2.2 L·mol^−1^·s^−1^ [78] and a pyruvate intracellular concentration of 0.1–0.5 mM (competitive with most thiols).

The toxicity of H_2_O_2_ can also be expressed indirectly. The reaction with bicarbonate leads to peroxymonocarbonate (HCO_4_^‒^) species that react approx. 300 times faster than H_2_O_2_ with thiols and sulfide (Figure 4) [79,80,81]. Only a few percent of H_2_O_2_ is present as peroxymonocarbonate in a physiological bicarbonate buffer since the reaction is an equilibrium (K = 0.32) [81]; it can be accelerated by carbonic anhydrase [79], thus enhancing the physiological relevance of the reaction. However, the most deleterious effect of H_2_O_2_ comes from its reaction with transition metals able to generate highly reactive radical species or activated metal complexes. The widely-known example is the production of hydroxyl radicals from hydrogen peroxide by the Fenton reaction (Figure 4), which involves iron ions as the metal catalyst. The reaction may be catalyzed by other redox competent metal ions (and, in this case, is called a “Fenton-like” reaction) as, for example, by copper complexed to the amyloid-beta peptide (Aβ) [82]. With the Fe^3+^ of heme proteins, the reaction of H_2_O_2_ is fast (k = 10^7^–10^8^ L·mol^−1^·s^−1^) [83] and give rise to Fe^4+^-oxoferryl porphyrin radical cation, able to transfer one electron to the surrounding protein [3,84], resulting in a formation of a protein radical that further evolves. The question of whether H_2_O_2_ is directly converted into HO^•^ radicals or whether intermediates of higher oxidation states of the metal are produced has been debated in the past years. Both are possibly involved, depending on circumstances, but they are all strong oxidative species and the products resulting from their reaction with a substrate should be similar. When metal ions are coordinated to a biological molecule, the reaction may be different from Fenton chemistry since metal-catalyzed oxidation (MCO) is site-directed. Such a case is observed, for instance, for protein, DNA or the Aβ peptide in iron- or copper-catalyzed oxidation in the presence of ascorbate [36,85,86].

### 3.4. Peroxyl Radicals, Hydroperoxides and Alkoxyl Radicals

Peroxyl radicals are secondary species generated by the addition of molecular oxygen on carbon-centered radicals ([O_2_] ≈ 2 × 10^−4^ mol·L^−1^ in aerated aqueous solution), whose rate constant usually range between 10^8^ and 10^9^ L·mol^−1^·s^−1^ [2]. They can also be produced in the absence of oxygen by metal-induced conversion of hydroperoxides [87]. Hydroperoxides are generated from peroxyl radicals by reaction with HO_2_^•^ or by H abstraction from another molecule, and may further react with HO_2_^•^ or a metal ion to generate alkoxyl radicals (Figure 4). The latter can also be generated from peroxyl radicals via a tetroxide. Peroxyl and alkoxyl radicals are oxidant species, with relatively high redox standard potentials of E°’(RO_2_^•^/RO_2_H) = 1.00 V and E°’(RO^•^/ROH) = 1.60 V, respectively [88].

Peroxyl radicals react faster than the superoxide anion with numerous biological substrates (DNA, lipids, proteins); rate constants [2] ranging from 10^2^ to 10^8^ L·mol^−1^·s^−1^. Even if they are much less reactive than the hydroxyl radical, they share some similarity in their mode of reaction as they are able to either be engaged in electron abstraction, H atom abstraction or addition on double-bonds (Figure 4) [89]. In the latter case, intra- or intermolecular reactions lead to the formation of the radical endoperoxide ROOR^•^ species. Peroxyl radicals with the α-hydroxyl or α-amino groups can also undergo rapid unimolecular elimination of HO_2_^•^/O_2_^•‒^, leading to carbonyl or imine group formation [2,90,91]. Peroxyl radicals ROO^•^ can undergo dimerization with other peroxyl radicals R’OO^•^ and yield tetroxide species ROO-OOR’; the reaction is also possible between peroxyl radicals and HO_2_^•^, as observed for thymine [59]. Tetroxide is an unstable species and their subsequent decomposition yields carbonyl groups and alcohol, accompanied by the loss of molecular oxygen [3].

Among the very diverse reactions that peroxyl radicals can initiate, some are of particular importance because they contribute to the degradation of cell membranes induced by lipid peroxidation. Once a carbon-centered radical has been generated on a fatty acid moiety, it reacts fast with molecular oxygen to yield a peroxyl radical, able to abstract an H atom from another fatty acid moiety to give birth to another carbon-centered radical. This H abstraction is facilitated by the proximity of the two fatty acid chains within the lipid bilayers of cell membranes. In such a condition, a chain reaction starts and is propagated by the R^•^ and RO_2_^•^ radicals. The chain reaction stops either when there are no more lipids, no more oxygen or when peroxide radicals react with a lipid-soluble antioxidant, such as α-tocopherol or carotenoids [92,93].

Primary and secondary alkoxyl radicals undergo a rapid 1,2-hydrogen shift, resulting in the generation of α-hydroxyalkyl radicals, in competition with the intramolecular 1,5-hydrogen shift and the formation of alcohol by intermolecular H abstraction [94,95]. In some cases, in particular, when a 1,2-hydrogen shift is not possible (tertiary alkoxyl radicals), β-fragmentation reactions occur and yield aldehydes and ketones [96] with relatively high rate constants in aqueous solutions (k > 10^6^ s^−1^) [97,98].

## 4. The Implication of ROS in Neurodegenerative Diseases

The high consumption of molecular oxygen and the high content of polyunsaturated fatty acid, strongly sensitive to peroxidation, make the brain a particularly vulnerable tissue to oxidative stress [99]. The latter is a modulator of neurodegenerative diseases (recently reviewed in Reference [7]). Peroxidation products of fatty acids are among the biomarkers of oxidative stress in neurodegenerative diseases such as Alzheimer’s disease (AD), Parkinson’s disease (PD) and amyotrophic lateral sclerosis (ALS), along with protein carbonylation and nitration, DNA and RNA oxidative damages [100,101,102,103,104]. Neurodegenerative diseases are commonly associated with abnormal protein aggregation. In AD, the Aβ peptide is found aggregated in senile plaques (composed of Aβ fibrils and metal ions) and hyperphosphorylated Tau in neurofibrillary tangles. In PD, aggregation of α-synuclein leads to Lewy bodies inclusions, while the aggregation of the huntingtin protein and copper/zinc superoxide dismutase are involved in Huntington’s disease (HD) and ALS, respectively. Such abnormal protein aggregation is able to induce oxidative stress via mitochondria dysfunction and ROS production [105,106,107], leading to chronic inflammation, and play an important role in neurodegeneration.

**Figure 5:**
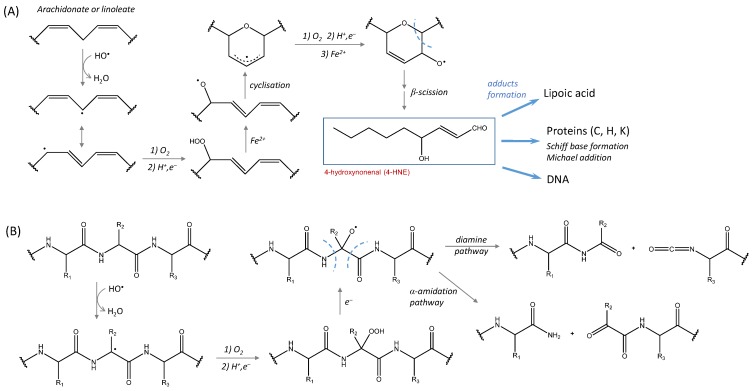
The direct involvement of ROS in lipid peroxidation and protein carbonylation. (**A**) the mechanism of 4-HNE formation from ROS-induced polyunsaturated omega-6 fatty acid peroxidation (from Pryor and Porter [125]); 4-HNE is able to form adducts with lipoic acid, proteins (C, H and K residues) and DNA bases; (**B**) ROS-induced protein carbonylation and cleavage (from Stadtman and Levine [43]).

In AD, an imbalance between the production of ROS and the reduced activity of enzymes responsible for ROS scavenging leads to oxidative damages on biomolecules, and on the Aβ peptide itself [108,109,110]. The link between oxidative stress and the amyloid beta peptide has been recently reviewed [111]. Because copper is present in relatively high levels in the brain and because of the ability of the Aβ peptide to chelate metal ions, Aβ-copper is a potential direct source of ROS in the presence of ascorbate and molecular oxygen. Reybier et al. [41] have shown that the superoxide anion is generated as an intermediate during H_2_O_2_ production by Aβ-copper. No direct link has yet been established between the production of ROS by Aβ-copper and oxidation of biological material in vivo. However, increased levels of lipids, protein and DNA oxidation have been reported to be associated with elevated levels of Aβ, whereas low Aβ-content brain regions do not present high concentrations of oxidative stress markers [112,113,114,115]. Lipid peroxidation is one of the events associated with AD, which might be involved in the phospholipid imbalance observed in the brain of AD patients [116,117]. Malondialdehyde (MDA) and 4-hydroxynonenal (4-HNE) are two aldehydes commonly found in high levels in AD brains [118]. The compound 4-HNE is toxic for neurons by causing apoptosis or by altering the microtubule structure [119,120], but is also prone to react with lipoid acid [121] and to form adducts with proteins (target amino acids are cysteine, histidine and lysine) [122] detected in AD brains [123]. In particular, adducts with Tau were found to modify its conformation and to favor neurofibrillary tangles formation [124]. The compound 4-HNE is generated by the non-enzymatic oxidation of polyunsaturated omega-6 fatty acids, such as arachidonate or linoleate. It is a direct consequence of the peroxidation of lipids by ROS since it is generated by the degradation of lipid hydroperoxides [125]—hydroperoxyoctadecadioenoate (HPODE) from linoleate or hydroperoxyeicosatetraenoate (HPETE) from arachidonate (Figure 5). F2- and F4-isoprostanes, which are generated by peroxidation of arachidonate, are other markers of oxidative stress in AD and found in elevated levels in the brain of AD patients [126,127,128].

Protein oxidation has been evidenced by high levels of carbonylated proteins in the brain areas the most involved in AD (i.e., hippocampus and parietal cortex) [114,129]. Several molecular mechanisms have been proposed for protein carbonylation, some of them being induced by direct ROS attacks and leading to protein cleavage via an alkoxyde radical formation (Figure 5). The target proteins are, among others, those involved in glucose metabolism and ATP synthesis [130], such as ATP synthase [131], pyruvate kinase, phosphoglucose mutase, α-enolase, malate dehydrogenase or glyceraldehyde-3-phosphate dehydrogenase (see Reference [132] for a review). Modifications detected include carbonylation, nitration and HNE-adducts formation. Like protein carbonylation, oxidative damages of DNA bases may result from a direct attack of ROS. Increased levels of 8-oxo-2-dehydroguanine, 8-hydroxyadenine and 5-hydroxyuracil have been reported in the temporal, parietal and frontal lobes of AD brains [133,134], along with 8-hydroxyguanine in the hippocampus of patients with preclinical stages of AD [135]. The high levels of oxidized DNA bases are detected in neurons where lipids and protein oxidation are also increased [136].

In PD, the involvement of ROS and oxidative stress might be one of the major factors causing the disease. Dopaminergic neurons of the substantia nigra, where the basal level of free radicals is important [137], are particularly sensitive to degeneration. Elevated levels of oxidized lipids and proteins have been detected in the substantia nigra of PD patients [138,139]. Additionally, an increase of 8-hydroxy-2′-deoxyguanosine and 8-hydroxyguanine levels, two markers of DNA oxidation, was observed [140]. As in AD, 4-HNE-modified proteins have also been detected in PD [141]. Thus, AD and PD share some similarities regarding the biomarkers of the oxidative stress detected. In PD, metal ion release (e.g., Fe^2+^) would be an important mechanism of neurodegeneration, through ROS production and dopamine oxidation. High levels of iron ions, in conjunction with the production of H_2_O_2_ via dopamine oxidation (enzymatically by monoamine oxidases, would lead to an overproduction of ROS and thus to oxidative stress conditions. Non-enzymatic oxidation of dopamine is involved in free radicals production and in elevation of free iron levels in dopaminergic cells [142,143,144]. Oxidative modification of proteins in PD may also have an impact on their propensity to aggregate. Surgucheva et al. [145] have shown that oxidation of γ-synuclein enhanced the formation of annular oligomers that accumulate in cells and that can initiate α-synuclein aggregation.

In other diseases such as ALS or HD, the link with oxidative stress is also evidenced, even if the mechanisms involved in their etiology are not fully understood. In ALS, oxidative stress is evidenced by elevated levels of MDA, 4-HNE, advanced oxidation protein products, isoprostanoids and 8-hydroxy-2′-deoxyguanosine [146,147,148,149,150]. Oxidative stress is also coupled to mitochondrial damages and dysfunction, each exacerbating the other, and to RNA dysmetabolism and unfolded protein aggregates formation [151,152]. As in other neurodegenerative diseases, copper and iron homeostasis is disturbed in ALS and elevated levels of these redox-competent metal ions could participate in ROS production [153]. Strong evidence exists also for early oxidative stress in HD, coupled with mitochondrial dysfunction, but it is still not clear whether oxidative stress is a cause or a consequence of HD. As for ALS, metal dyshomeostasis, evidenced by high levels of iron and copper in post-mortem brain tissues of HD patients [154], would participate in ROS production via Fenton chemistry. Increases of nuclear and mitochondrial DNA 8-hydroxy-2′-deoxyguanosine were detected in the blood and serum of HD patients [155,156], along with DNA double-strand breaks, a potential result of free radical damage [157,158]. Lipid peroxidation (high levels of MDA and cytoplasmic lipofuscin), protein carbonylation and nitration (an increase of 3-nitrotyrosine levels) are also observed in HD [159,160].

## 5. Concluding Remarks

An important feature shared by most of the neurodegenerative diseases is the presence of oxidative damages that link them to oxidative stress. The latter is supposed to be an early event in the etiology of some diseases since the biomarkers of oxidation appear early in their development [132]. An overproduction of ROS is considered to have a major contribution in oxidative damages undergone by biomolecules, including lipids, proteins and DNA. The intrinsic chemical properties of ROS make them formidable weapons against most biomolecules. Among them, because of its diffusion-controlled reactivity with most of the biological material, HO^•^ may be considered as a nuclear weapon compared to O_2_^•‒^ and H_2_O_2_. These last two react directly only with few specific targets (e.g., SOD and catalase). However, they are strong deleterious species because of (i) their ability to be engaged in Fenton and Haber-Weiss reactions, two metal-catalyzed reactions that lead to HO^•^ production, (ii) their lack of direct reactivity which gives them the possibility to spread to areas where metal levels are high. So, most of the time, final oxidative damages on biomolecules could be considered as resulting from HO^•^ attacks. This could particularly apply to neurodegenerative diseases where metal dyshomeostasis takes place and where elevated levels of redox-competent metal ions—such as iron or copper—are observed. In this context, better control of ROS homeostasis would be important for neuron survival. This could be achieved, among others, by developing antioxidant-based strategies. This is the reason why many studies have focused and are still focusing on possible therapeutic approaches based on antioxidant strategies, either by the administration of antioxidant in the form of plant extracts or nutraceuticals [161,162] or by reinforcing the antioxidant defense system in vivo [163]. Antioxidant therapy-based strategies to fight against neurodegenerative diseases have shown promising results in preclinical trials but only a few clinical trials have been conducted and the benefit of such a therapy is still under debate [164]. In this context, and because most of the mechanisms underlying the etiology of neurodegenerative diseases have still not been elucidated, all efforts to better understand, through basic research, the causes of disease development will increase the global knowledge and will help to develop novel therapeutic strategies.
